# αII-spectrin and βII-spectrin do not affect TGFβ1-induced myofibroblast differentiation

**DOI:** 10.1007/s00441-018-2842-x

**Published:** 2018-05-03

**Authors:** Bram Piersma, Olaf Y. Wouters, Ruud A. Bank

**Affiliations:** University Medical Center Groningen, Department of Pathology and Medical Biology, University of Groningen, Hanzeplein 1, 9713 GZ Groningen, The Netherlands

**Keywords:** Spectrin, Fibroblast, TGFβ1, Physiological stiffness, Mechanosensing

## Abstract

Mechanosensing of fibroblasts plays a key role in the development of fibrosis. So far, no effective treatments are available to treat this devastating disorder. Spectrins regulate cell morphology and are potential mechanosensors in a variety of non-erythroid cells, but little is known about the role of spectrins in fibroblasts. We investigate whether αII- and βII-spectrin are required for the phenotypic properties of adult human dermal (myo)fibroblasts. Knockdown of αII- or βII-spectrin in fibroblasts did not affect cell adhesion, cell size and YAP nuclear/cytosolic localization. We further investigated whether αII- and βII-spectrin play a role in the phenotypical switch from fibroblasts to myofibroblasts under the influence of the pro-fibrotic cytokine TGFβ1. Knockdown of spectrins did not affect myofibroblast formation, nor did we observe changes in the organization of αSMA stress fibers. Focal adhesion assembly was unaffected by spectrin deficiency, as was collagen type I mRNA expression and protein deposition. Wound closure was unaffected as well, showing that important functional properties of myofibroblasts are unchanged without αII- or βII-spectrin. In fact, fibroblasts stimulated with TGFβ1 demonstrated significantly lower endogenous mRNA levels of αII- and βII-spectrin. Taken together, despite the diverse roles of spectrins in a variety of other cells, αII- and βII-spectrin do not regulate cell adhesion, cell size and YAP localization in human dermal fibroblasts and are not required for the dermal myofibroblast phenotypical switch.

**Electronic supplementary material:**

The online version of this article (10.1007/s00441-018-2842-x) contains supplementary material, which is available to authorized users.

## Introduction

Chronic organ injury often results in the development of fibrosis: an excessive production, post-translational modification and stiffening of extracellular matrix (ECM) components (Rockey et al. 2015). Pathological stiffening of the ECM creates a pro-fibrotic feedback loop (Parker et al. 2014) but how mechanical cues are transduced to change cell function and fate remains incompletely understood. Driving the fibrotic response are activated fibroblasts or pericytes that acquire the myofibroblast phenotype, which is characterized by a well-developed endoplasmic reticulum and an extensive contractile actomyosin cytoskeleton (Klingberg et al. 2013). Decades of research have been devoted to the contractile apparatus in the regulation of the myofibroblast phenotype. More recently, structural proteins belonging to the spectrin family were found to act as functional adaptors between the actomyosin cytoskeleton and the plasma membrane and are thought to regulate transduction of mechanical signals (Liem 2016; Stankewich et al. 2011).

Spectrins form a major component of the cytoskeleton at the membrane-cytoskeleton interface (Bennett 1990a; Sormunen 1993) and play an important role in maintaining cellular integrity (Bennett and Baines 2001). Spectrins form tetrameric flexible heterodimers, which contain two alpha and two beta subunits (Dubreuil et al. 1989; MacDonald and Cummings 2004) and have been evolutionary conserved in species as different as echinoderms (Fishkind et al. 1987), *Sophophora* (Bennett 1990a; Deng et al. 1995; Dubreuil et al. 1987, 1990), birds (Wasenius et al. 1989) and humans (Bennett 1990b; Leto et al. 1988; Sevinc and Fung 2011). They were first discovered in metazoan erythrocytes where they support the membrane cytoskeleton (Bennett 1990a, 1990b; Bennett and Baines 2001). In erythrocytes, two different spectrin genes are found, *SPTA1* (αI-spectrin) and *SPTB1* (βI-spectrin). Both subtypes are uniquely expressed in erythrocytes and thus not found in other cell types (Wasenius et al. 1989). More recently, other spectrin proteins were identified in non-erythrocyte cells (Bennett 1990a; Dubreuil et al. 1990; Moon and McMahon 1990). *SPTAN1* encodes several isoforms of the non-erythrocyte αII-spectrin polypeptide that are generated through alternative splicing. In addition, non-erythrocyte β-spectrins are encoded by four similar genes: *SPTBN1* (βII-spectrin), *SPTBN2* (βIII-spectrin), *SPTBN4* (βIV-spectrin) and *SPTBN5* (bV-spectrin (βHeavy)). Here, we focus on αII-spectrin and βII-spectrin, since they have been reported to provide mechanical stability and maintaining cell integrity, plasma membrane stability and morphology—key features of cellular mechanosensing (Bialkowska 2005; Machnicka et al. 2012; Metral et al. 2009; Stankewich et al. 2011). Furthermore, αII-spectrin and βII-spectrin regulate cell adhesion (Metral et al. 2009) and cell spreading (Bialkowska 2005; Meriläinen et al. 1993; Stankewich et al. 2011) and contain domains that function in protein sorting, vesicle trafficking and endocytosis (Bialkowska 2005; Devarajan et al. 1997; Kamal et al. 1998).

The functional domain in the αII-spectrin subunit is the highly conserved Src Homology 3 (SH3) domain (Musacchio et al. 1992), which initiates Rac activation during initial cell adhesion (Bialkowska 2005). In addition, αII-spectrin contains a calmodulin binding site (Bennett 1990a; Dubreuil et al. 1987), which might be involved in cell contraction and migration. Furthermore, αII-spectrin is reported to be involved in regulation of actin dynamics (Bialkowska 2005) and βII-spectrin is involved in TGFβ1 signaling, where it functions as a SMAD adaptor protein (Baek et al. 2011; Kitisin et al. 2007; Tang et al. 2003). Additionally, spectrins associate with, as well as regulate, Yes-associated protein 1 (YAP) (Fletcher et al. 2015; Wong et al. 2015). YAP is a mechanosensitive transcriptional co-factor of genes involved in proliferation and suppression of apoptotic genes (Calvo et al. 2013; Dupont et al. 2011; Janmey et al. 2013) and is regulated by both Hippo and TGFβ1 signaling (Liu et al. 2015; Piersma et al. 2015a, b). Whether spectrins play a role in the myofibroblast phenotypical switch remains unknown. Here, we study the role of αII-spectrin and βII-spectrin in stiffness-induced cell spreading and adhesion, YAP translocation and wound closure in human dermal fibroblasts. Furthermore, we examine the role of αII-spectrin and βII-spectrin in TGFβ1-induced myofibroblast differentiation.

## Materials and methods

### Reagents and antibodies

Reagents were as follows: human plasma fibronectin (20 μg/mL, F1056; Sigma-Aldrich, Munich, Germany), human recombinant TGFβ1 (10 ng/mL, 100-21C; Peprotech, London, UK), αII-spectrin siRNA (25 ng/cm^2^, EHU093741; Sigma-Aldrich), βII-spectrin siRNA (25 ng/cm^2^, EHU081451; Sigma-Aldrich), *Renilla* luciferase siRNA (25 ng/cm^2^, EHURLUC; Sigma-Aldrich), Alexa647-labeled streptavidin (8 μg/mL, S32357; Thermo Fisher Scientific, Landsmeer, The Netherlands), TRITC labeled-Phalloidin (100 nM, P1951; Sigma-Aldrich). Antibodies used: mouse anti-αII-spectrin (2 μg/mL, sc-376849; Santa Cruz, Dallas, USA), mouse anti-βII-spectrin (2 μg/mL, sc-376487; Santa Cruz), mouse anti-αSMA (0.28 μg/mL, M0851; DAKO; Glostrup, Denmark), mouse anti-collagen type I (1 μg/mL, ab90395; Abcam, Cambridge, UK), mouse anti-vinculin (9.3 μg/mL, V9131; Sigma-Aldrich).

### Cell manipulations

Before the onset of experiments, normal adult human dermal fibroblasts (CC-2511, nHDF-Ad-Der; Lonza, Basel, Switzerland) were propagated in DMEM (12-604F; Lonza) supplemented with 2 mM l-glutamine, 50 U/L penicillin/streptomycin and 10% FCS. For protein knockdown experiments, cells were seeded at 15.000 cells/cm^2^ and transfected with siRNA using Lipofectamine RNAiMax reagent (Thermo Fischer Scientific) and incubated for 72 h in DMEM supplemented with 1.5 mM l-glutamine, 38 U/L penicillin/streptomycin and 7.5% FCS. siRNA targeting *Renilla* luciferase was used as negative control. After the transfection period, cells were cultured for an additional 96 h in DMEM containing 0.5% FCS supplemented with 2 mM l-glutamine and 50 U/L penicillin/streptomycin to ensure elimination of the spectrin proteins, as they are relatively long-lived proteins. Efficiency of knockdown was subsequently determined by means of qPCR and immunofluorescence. For cell adhesion, cell spreading and YAP translocation studies, cells were reseeded on fibronectin-functionalized polyacrylamide gels for 24 h. Cell spreading was determined by measuring cell surface area with Nuance FX software (Perkin Elmer, Groningen, The Netherlands). Cell adherence was determined by quantifying the number of cells in 25 FOVs. YAP translocation was measured by means of immunofluorescence.

For myofibroblast differentiation experiments and the wound healing assay, the trypsinized cells were reseeded on polystyrene culture wells (for mRNA measurements or wound healing) or slides (for immunostaining); cultured in DMEM containing 0.5% FCS, 2 mM l-glutamine, 50 U/L penicillin/streptomycin and 0.17 mmol/L ascorbic acid (A8960; Sigma-Aldrich); and supplemented with or without TGFβ1 (10 ng/mL) for 72 h. For the wound healing assay, IBIDI inserts were removed after 48 h, leaving another 24 h for the cells to repopulate the wound area.

### Fibronectin-functionalized polyacrylamide hydrogels

To determine the role of spectrins in cell adhesion and spreading, cells were seeded on fibronectin-functionalized polyacrylamide hydrogels with an elastic modulus of either 2 or 50 kPa. Polyacrylamide hydrogels were prepared as described previously (Wouters et al. 2016). In brief, gels were prepared between a chemically modified glass plate and coverslip. The glass plate was cleaned by immersion in 99.9% ethanol for 15 min and treated with dichlorodimethylsilane to avoid polyacrylamide interactions. Glass coverslips were treated with 0.5% trimethoxypropylmethacrylate in 99.1% ethanol, which was activated using 0.3% glacial acetic acid to facilitate covalent adhesion of polyacrylamide hydrogels. Differences in stiffness (elastic modulus) were obtained by varying the ratio between acrylamide and bisacrylamide and the Young’s modulus was validated by means of Atomic Force Microscopy (AFM). Hydrogel polymerization was initiated with TEMED and APS. To functionalize the surface of the hydrogels, they were overlaid with 2 mg/ml l-DOPA (in 10 mM Tris) and incubated for 30 min. Next, l-DOPA was washed off and hydrogels were functionalized with 20 μg/mL plasma fibronectin for 2 h at 37 °C.

### RNA isolation, cDNA synthesis and qRT-PCR

To obtain total RNA, the FavorPrep Tissue Total RNA Purification Mini Kit (FATRK; Favorgen Biotech Corp., Taiwan) was used in accordance with the manufacturer’s protocol. RNA concentration and purity were determined by UV spectrophotometry (NanoDrop Technologies, Wilmington, NC). To assess gene expression, the RNA was reverse transcribed using the First Strand cDNA synthesis kit (Thermo Fisher Scientific) using random hexamer primers in accordance with the manufacturer’s instructions. Gene expression quantification was performed using qRT-PCR analysis and SYBR Green Supermix (Roche, Basel, Switzerland). The thermal cycling conditions were 2 min at 95 °C (enzyme activation), followed by 15 s at 95 °C, 30 s at 60 °C, and 30 s at 72 °C (40 cycles). All qPCRs were performed with a ViiA™ 7 Real-Time PCR System (Applied Biosystems, Foster City, CA, USA). Melting curve analysis was performed to verify the absence of primer dimers. Analysis of the data was performed using ViiA7™ Real-Time PCR System Software v1.2.4 (Applied Biosystems). Primer sequences are provided in Table 1.

**Table 1:** qPCR primers

Gene name	Forward primer	Reverse primer
ACTA	CTGTTCCAGCCATCCTTCAT	TCATGATGCTGTTGTAGGTGGT
COL1A1	GCCTCAAGGTATTGCTGGAC	ACCTTGTTTGCCAGGTTCAC
SPTAN1	AAGAAGCACGAAGACTTTGAGAA	TGGTTGCAAATTCATCTAATGC
SPTBN1	CCCAGCAGGACAAACTCAAC	GGCATCCTTCTTCCTGTCAA
SPTBN2	AGGTCGTGCAGCAGAGGT	GTAACTGCTCGGCAATGTCA
SPTBN4	GAGCTGGCTGAATGAGAACC	GGCAGCTCATACCCAAAGTT
SPTBN5	CCACACAAATCCAACGACAG	TCCTGCAGAAAACTGCACAC
YWHAZ	GATCCCCAATGCTTCACAAG	TGCTTGTTGTGACTGATCG

### Immunofluorescence

For spectrin immunofluorescence, cells cultured for 7 days were fixed in 4% PFA and incubated with 10% goat serum in PBS for 1 h. Primary antibodies were incubated in PBS + 2.2% BSA at room temperature (RT) for 2 h. For YAP immunofluorescence, cells were permeabilized with 0.5% Triton X-100 and subsequently incubated with PBS 10% goat serum RT for 1 h. Primary antibodies were incubated in PBS + 0.1% Triton X-100 and 2.2% BSA at 4 °C for 16 h. For α-smooth muscle actin and collagen immunofluorescence, methanol/acetone (1:1) fixed cells were incubated with 10% goat serum for 1 h and primary antibodies were incubated in PBS + 2.2% BSA at RT for 2 h. For all immunofluorescence, secondary antibodies were diluted in PBS + 2.2% BSA at RT for 1 h and subsequently incubated with Alexa647-labeled streptavidin in PBS containing 4′,6-diamidino-2-phenylindole (DAPI, 1:5000, 10236276001; Roche) for 30 min. Actin was visualized by incubation with TRITC labeled-Phalloidin in PBS for 30 min. Between incubations, cells were washed thrice with PBS containing 0.5% Tween-20. Slides were mounted in Citifluor (Agar Scientific, Stansted, UK) and used for immunofluorescence microscopy.

### Statistics

All data are represented as means ± SD of at least three independent experiments and were analyzed by GraphPad Prism Version 7.01 for Windows (GraphPad Software, Inc., La Jolla, CA, USA) by either one-way or two-way ANOVA followed by Bonferroni post hoc analysis.

## Results

### αII- and βII-spectrin do not influence cell adhesion

**Fig. 1:**
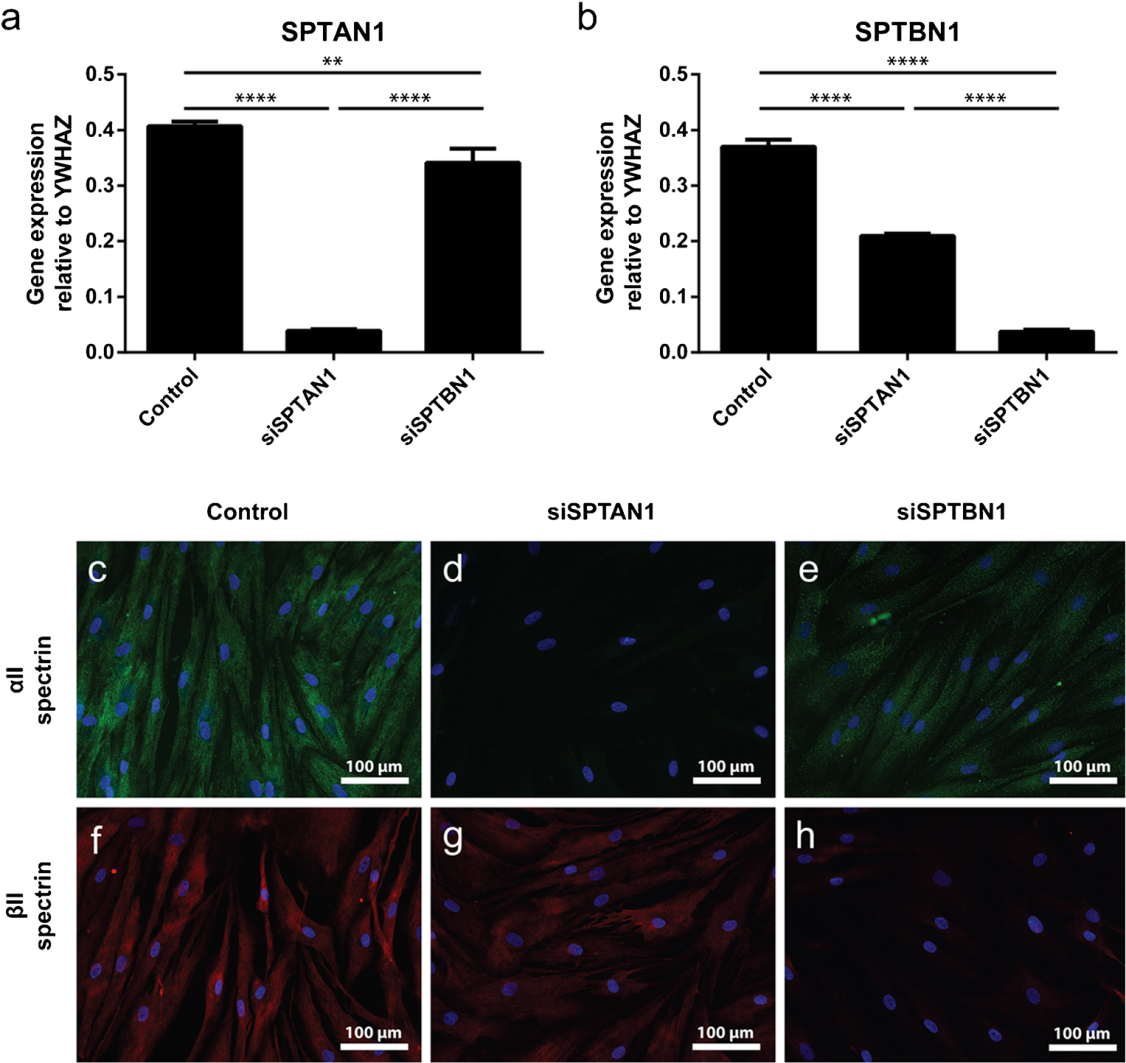
αII-spectrin and βII-spectrin knockdown with esiRNA. (a, b) mRNA expression of αII-spectrin (*SPTAN1*) and βII-spectrin (*SPTBN1*) 7 days after esiRNA transfection. One-way ANOVA; **p < 0.01, ****p < 0.0001. (c–h) Representative immunofluorescent images of αII-spectrin and βII-spectrin 7 days after esiRNA transfection. Original magnification × 200

**Fig. 2:**
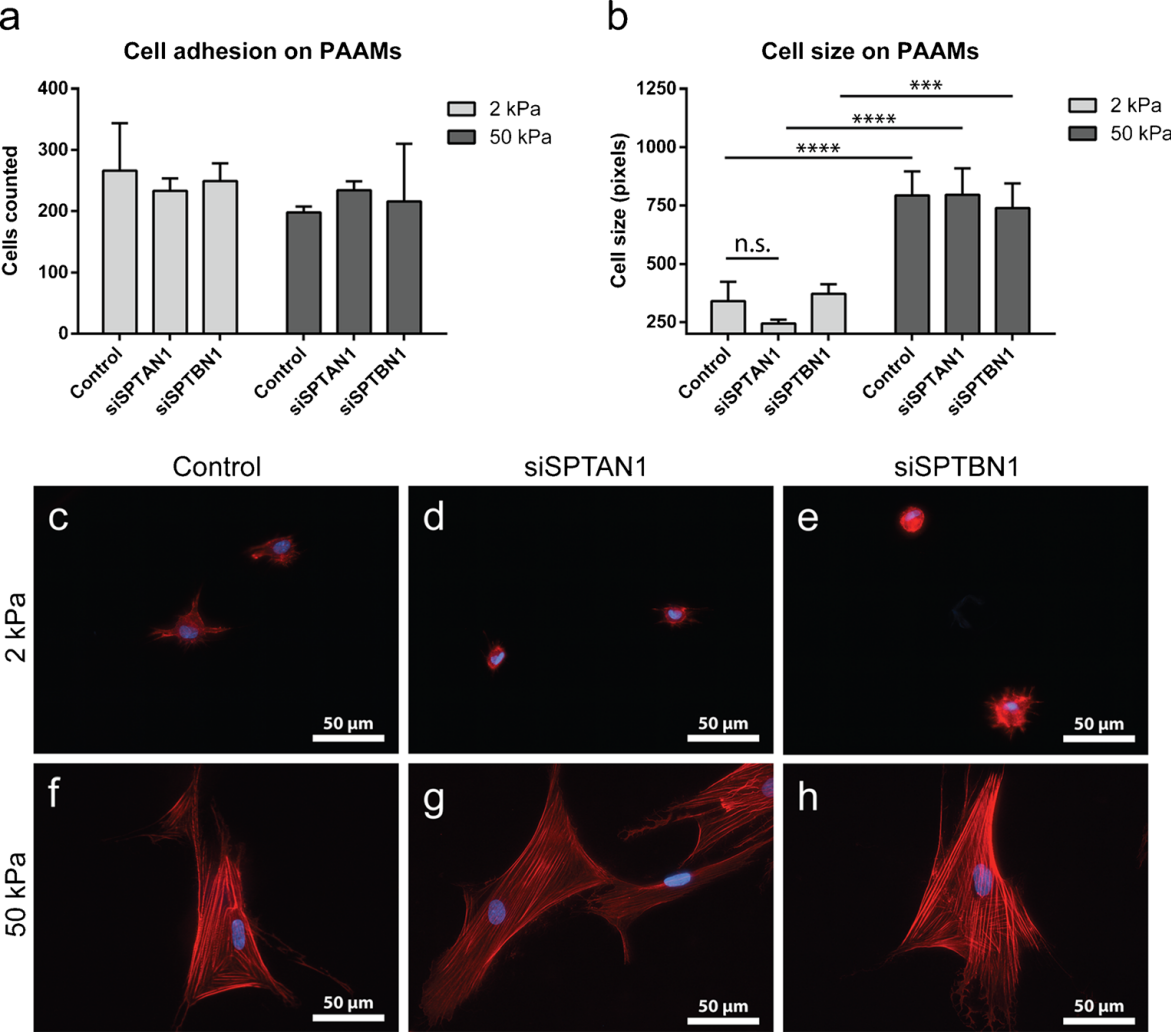
αII- and βII-spectrin do not mediate fibroblast spreading and adhesion. (a) Cell adhesion on 2 and 50 kPa polyacrylamide hydrogels. (b) Effect of hydrogel stiffness on cell spreading. Two-way ANOVA; ***p < 0.001, ****p < 0.0001. (c–h) F-actin (phalloidin) and nuclear (DAPI) staining to visualize cell size and cell adhesion. Original magnification × 400. DAPI, 4′,6-diamidino-2-phenylindole; kPa, Kilo Pascal; PAAM, polyacrylamide

In order to elucidate the role of spectrins on fibroblast behavior, we performed siRNA-mediated knockdown. We found that both αII- and βII-spectrin have a long half-life; we only observed > 90% gene expression knockdown 168 h (7 days) after transfection (Fig. 1a, b) and also observed knockdown at the protein level (Fig. 1c–h). Interestingly, βII-spectrin siRNA knockdown also decreased the expression of αII-spectrin about twofold. Next, we investigated whether αII- and βII-spectrin have an effect on cell adhesion (Fig. 2a) by seeding cells on either soft (2 kPa) or stiff (50 kPa) fibronectin-coated substrates. Cell adhesion did not differ between 2 and 50 kPa in either the control cells or the αII-spectrin- and βII-spectrin-deficient cells.

### Cell spreading on soft and stiff substrates is independent of αII- and βII-spectrin

The morphological and cytoskeletal changes of fibroblasts are well documented for cells cultured on fibronectin-coated surfaces with stiffness ranging from 2 to 50 kPa. When grown in sparse culture with no cell-cell contacts, fibroblasts show an abrupt change in spread area that occurs at a stiffness range above 3 kPa (Yeung et al. 2005). We indeed observed major differences in cell size (spreading) between 2 and 50 kPa gels: cells cultured on 2 kPa were markedly smaller than cells cultured on 50 kPa (Fig. 2b–h). This was the case both for control cells as for αII-spectrin- or βII-spectrin-deficient cells but we observed no significant differences in cell size between the spectrin-deficient cells and the control group. These data suggest that αII- and βII-spectrin do not affect the stiffness-dependent changes in cell size of dermal fibroblasts.

### αII- and βII-spectrin do not regulate YAP localization

**Fig. 3:**
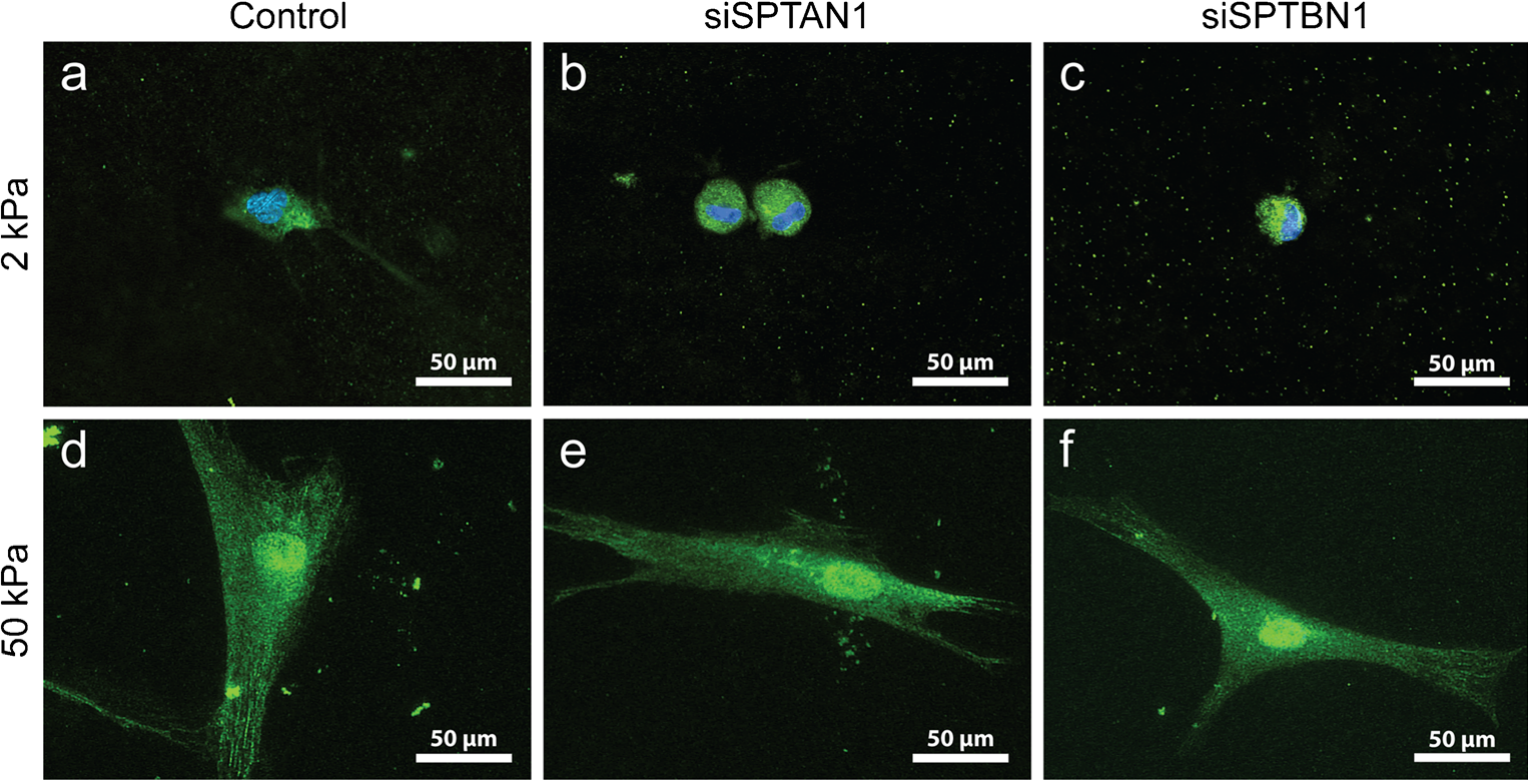
YAP nuclear accumulation is independent from αII- and βII-spectrin. (a–f) Yes-associated protein 1 (YAP; green) localization in spectrin KD fibroblasts cultured on either 2 or 50 kPa polyacrylamide hydrogels. Nuclei are stained with DAPI. Original magnification × 400. DAPI, 4′,6-diamidino-2-phenylindole; kPa, Kilo Pascal

YAP is a mechanosensitive transcriptional co-activator that has been shown to govern the phenotypical switch to myofibroblasts and accumulates in the nucleus on increased stiffness of the ECM (Piersma et al. 2015a, b; Szeto et al. 2016). One of the mechanisms of YAP nuclear accumulation involves polymerization of actin monomers into stress fibers (Aragona et al. 2013; Das et al. 2016; Dupont et al. 2011). Recently, αII- and βII-spectrin were shown to regulate cytoplasmic retention of YAP in stretched epithelial cells, by interacting with and activating Hippo signaling at the plasma membrane (Fletcher et al. 2015). Because fibroblasts and myofibroblasts rely heavily on their contractile cytoskeleton and are known for their ability to spread over great distances, we investigated the effects of substrate stiffness and the presence of αII- and βII-spectrin on YAP localization. We observed major differences in YAP localization between 2 and 50 kPa hydrogels (Fig. 3): on 2 kPa, almost all cells displayed cytoplasmic retention of YAP (Fig. 3a–c), while on 50 kPa, the majority of cells showed both nuclear and cytoplasmic localization of YAP (Fig. 3d–f). However, we observed no differences in YAP localization between spectrin-deficient cells and the control cells, suggesting that spectrins do not regulate YAP localization in dermal fibroblasts.

### αII- and βII-spectrin do not regulate fibroblast migration and wound healing

**Fig. 4:**
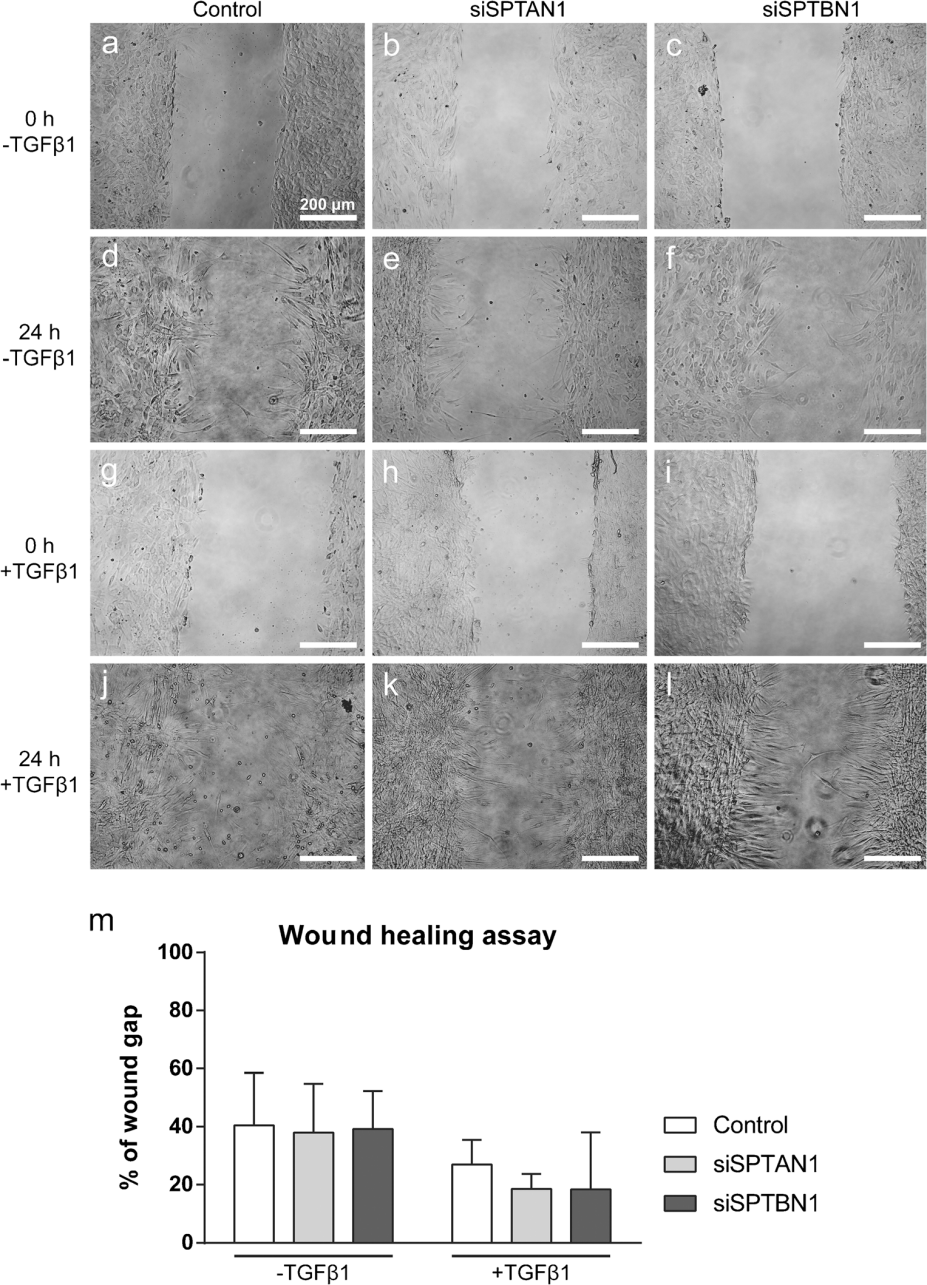
αII- and βII-spectrin do not influence wound gap closure. (a–l) Cells seeded at high density were left to repopulate the wound gap for 24 h in the presence or absence of TGFβ1 stimulation. (m) Quantification of panels a–l. Original magnification × 100. TGF, transforming growth factor

Others showed β_H_-spectrin to be involved in epithelial cell migration in *Sophophora* (Urwyler et al. 2012). Therefore, we asked whether spectrins are necessary for fibroblasts wound closure in vitro. We mimicked wound closure by means of IBIDI inserts and found no differences in wound repopulation in fibroblasts stimulated with or without TGFβ1 (Fig. 4a–l). Moreover, knockdown of spectrins did not affect the population rate of the wound area (Fig. 4(m)).

### αII- and βII-spectrin do not affect the myofibroblast phenotype

**Fig. 5:**
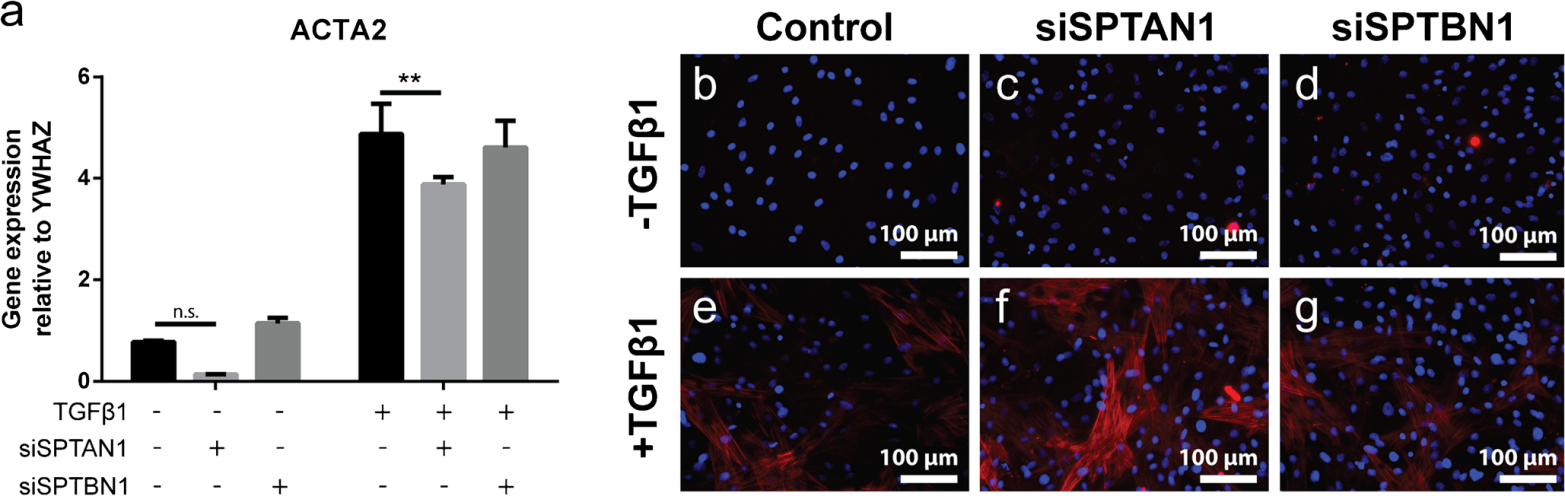
Spectrins do not regulate αSMA stress fiber formation. (a) mRNA expression of *ACTA2* (αSMA) after 4 days of stimulation with TGFβ1 on αII-spectrin (*SPTAN1*) and βII-spectrin (*SPTBN1*) KD cells. Two-way ANOVA; **p < 0.01. (b–g) Representative immunofluorescent images of α-smooth muscle actin stress fiber formation in αII-spectrin (*SPTAN1*) and βII-spectrin (*SPTBN1*) KD cells. Nuclei are stained with DAPI. Original magnification × 200. αSMA, α smooth muscle actin; DAPI, 4′,6-diamidino-2-phenylindole; TGF, transforming growth factor

Myofibroblasts play an important role in both regular wound healing as well as dysregulated wound healing, the latter resulting in fibrosis. αSMA stress fiber formation is an important hallmark of the myofibroblast phenotype, which can be induced by TGFβ1. We indeed observed an increase in *ACTA2* mRNA levels (Fig. 5a) and formation of αSMA stress fibers on TGFβ1 exposure (Fig. 5b–g). However, we observed no differences in the percentage of αSMA-positive cells between spectrin-deficient and control cells, suggesting that spectrins are not required for TGFβ1-induced formation of αSMA stress fibers. This was reflected in the mRNA levels of *ACTA2* between spectrin-deficient and control cells, although knockdown of βII-spectrin resulted in slightly lower *ACTA2* mRNA levels in TGFβ1-stimulated cells. Interestingly, knockdown of αII-spectrin had a significant effect on *ACTA2* expression in non-stimulated cells: the mRNA level was markedly lower (Fig. 5a).

### Collagen deposition is spectrin independent

**Fig. 6:**
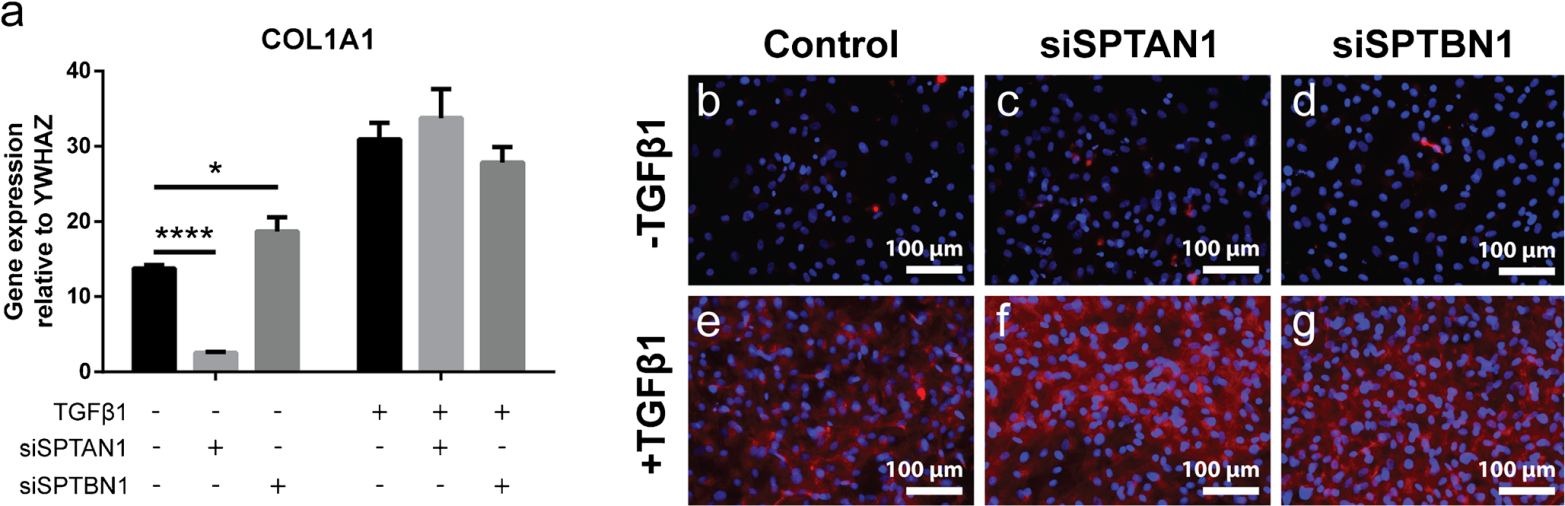
Spectrins do not regulate collagen type I synthesis. (a) mRNA expression of *COL1A1* after 4 days of stimulation with TGFβ1 on αII-spectrin (*SPTAN1*) and βII-spectrin (*SPTBN1*) KD cells. Two-way ANOVA; *p < 0.05, ****p < 0.001. (b–g) Representative immunofluorescent images of collagen type I deposition in αII-spectrin (*SPTAN1*) and βII-spectrin (*SPTBN1*) KD cells. Nuclei are stained with DAPI. Original magnification × 200. ANOVA, analysis of variance; DAPI, 4′,6-diamidino-2-phenylindole; TGF, transforming growth factor

To determine if another hallmark function of myofibroblasts, namely the increased synthesis of collagen type I, is regulated by spectrins, we determined mRNA levels and collagen deposition. We indeed observed large differences between cells stimulated with or without TGFβ1. The increase in *COL1A1* mRNA levels (Fig. 6a) was accompanied by an increase in collagen deposition (Fig. 6(b–g). However, αII- and βII-spectrin knockdown did not affect mRNA levels of *COL1A1* in TGFβ1-stimulated cells (Fig. 6a) or the deposition of collagen type I (Fig. 6b–g). Interestingly, knockdown of βII-spectrin had a major effect on *COL1A1* expression in non-stimulated cells: the mRNA level was markedly lower (Fig. 6a).

### αII- and βII-spectrin are not necessary for focal adhesion assembly

**Fig. 7:**
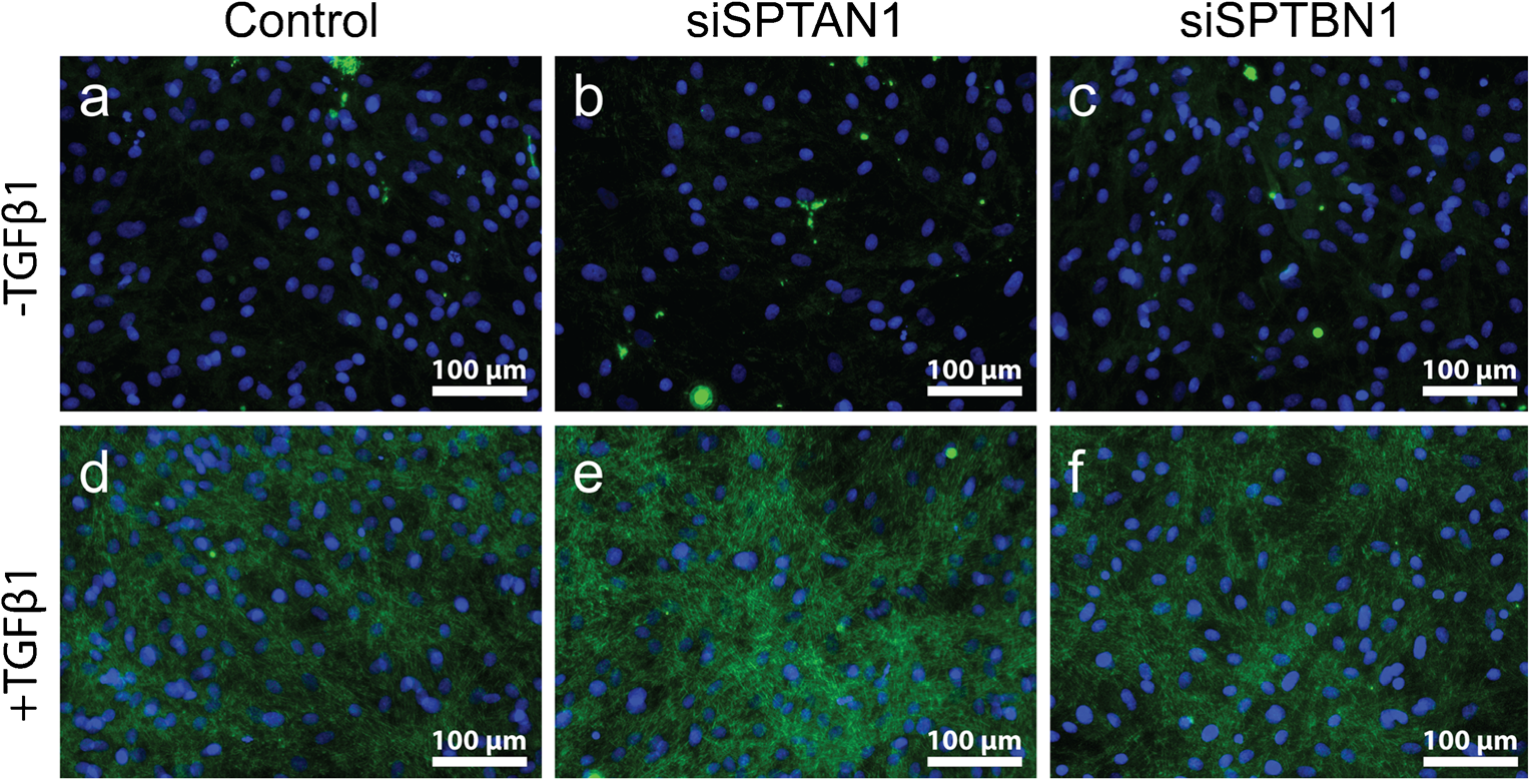
αII- and βII-spectrin do not control vinculin adhesions. (a–f) Representative immunofluorescent images of vinculin after 4 days of stimulation with TGFβ1 in αII-spectrin (*SPTAN1*) and βII-spectrin (*SPTBN1*) KD cells. Original magnification × 200. DAPI, 4′,6-diamidino-2-phenylindole; TGF, transforming growth factor

We determined the formation of focal adhesions by means of vinculin staining as a function of TGFβ1. As expected, a major increase in focal adhesions was observed under the influence of TGFβ1 (Fig. 7a–f). However, we did not observe any differences in focal adhesion formation between spectrin-deficient and control cells.

### TGFβ1 attenuates SPTAN1 and SPTBN1 expression

**Fig. 8:**
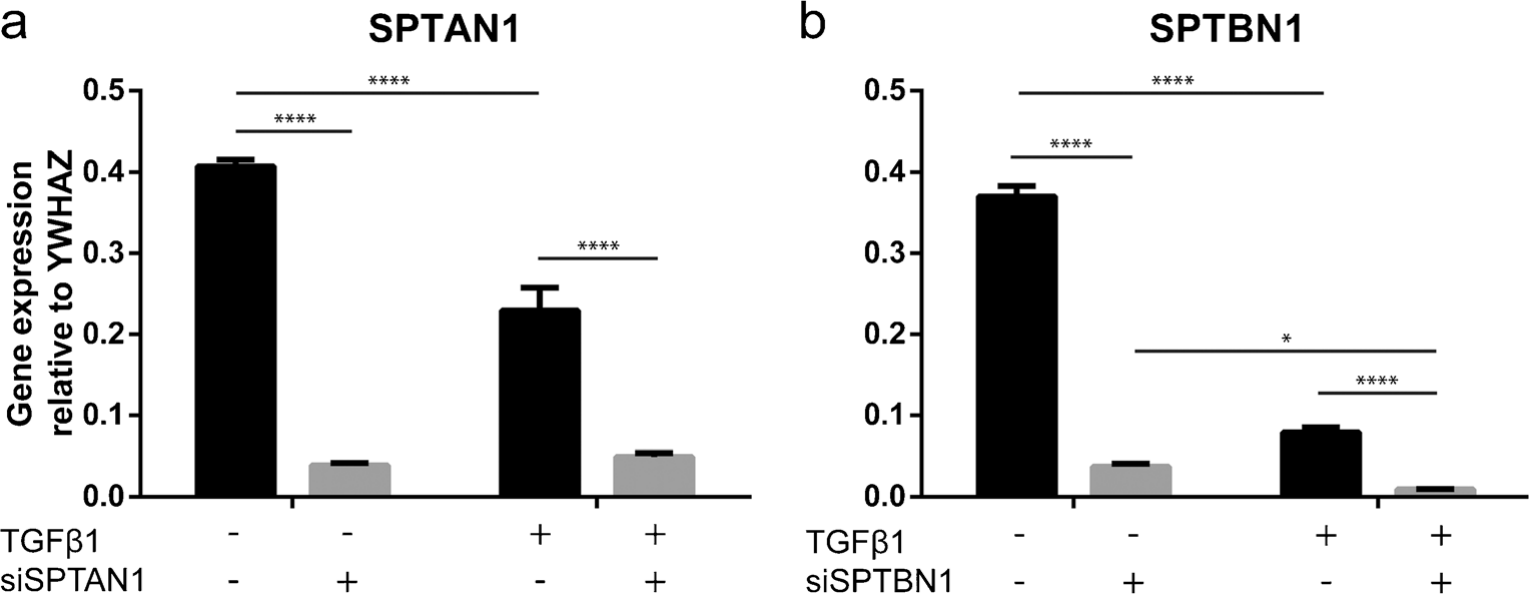
TGFβ1 stimulation decreases αII-spectrin (*SPTAN1*) and βII-spectrin (*SPTBN1*) gene expression. **a**, **b** mRNA expression of αII-spectrin (*SPTAN1*) and βII-spectrin (*SPTBN1*) after TGFβ1 stimulation. Two-way ANOVA; *p < 0.05, ****p < 0.0001. ANOVA, analysis of variance; TGF, transforming growth factor

Since we did not observe differences in myofibroblast parameters between control and cells KD for spectrins, we wondered what happens with endogenous *SPTAN1* and *SPTBN1* mRNA levels when cells are stimulated with TGFβ1. Interestingly, TGFβ1 stimulation had a direct negative effect on *SPTAN1* and *SPTBN1* gene expression, as incubation with TGFβ1 resulted in significantly lower mRNA levels of *SPTAN1* and *SPTBN1* (Fig. 8a, b). The effect of TGFβ1 on *SPTBN1* was more pronounced than for *SPTAN1*. To evaluate any possible compensatory mechanisms of αII- and βII-spectrin knock down, we interrogated the expression of three alternative spectrins, *SPTBN2*, *SPTBN4*, and *SPTBN5*. Interestingly, we found that knock down of αII-spectrin and βII-spectrin reduced the expression of SPTBN2/SPTBN4 and SPTBN4, respectively (Supplemental Fig. 1). Additionally, TGFβ1 exposure further decreased expression of all three alternative spectrins, which is in line with the expression pattern of *SPTAN1* and *SPTBN1*.

## Discussion

Although much is known about the function of spectrins in erythrocytes, less detailed information is available regarding the function of spectrins in non-erythroid cells, including fibroblasts. Since spectrins regulate cell morphology and are potential mechanosensors, we investigated whether αII- and βII-spectrin are required for the phenotypic properties of adult human dermal (myo)fibroblasts.

We first determined the effect of αII- and βII-spectrin on cell adhesion and cell spreading on 2 and 50 kPa gels and noticed that αII- and βII-spectrin do not regulate the adhesion or spreading of adult dermal fibroblasts, nor did we find morphological differences. This is of interest, as knockdown of spectrins results in major changes in shell shape in a variety of cell types. Mouse embryonic fibroblasts devoid of *SPTBN1* obtained at E14.5 showed an impaired cell spreading and had a more rounded and spiky appearance. In addition, a reduction in cell proliferation was observed (Stankewich et al. 2011). Unfortunately, *SPTBN1* null mice are embryonic lethal, so the functions of βII-spectrin in adult fibroblasts are not known. The discrepancy between our data obtained with adult cells compared with the above mentioned embryonic cells suggests that there could be age-related differences regarding the role of spectrins in cell shape of a specific cell type. This is substantiated by the observation that no changes in cell shape or morphology were observed in embryonic epithelial cells of *SPTBN1* knockdown mice (Stankewich et al. 2011), whereas major cell shape differences were observed in adult epithelial cells of humans (Kizhatil et al. 2007).

Next, we determined whether αII- and βII-spectrin have an effect on the translocation of YAP as a function of stiffness and cell spreading. We mimicked cell spreading by sparsely culturing the fibroblasts on 2 and 50 kPa gels. As expected, YAP was largely localized in the cytoplasm in cells cultured at 2 kPa and was abundantly localized in the nucleus in cells cultured at 50 kPa. Deficiency of αII- or βII-spectrin did not change the translocation pattern of YAP. It has been shown that αII-spectrin and βII-spectrin have a mechanosensory function in the Hippo pathway in epithelial cells (Fletcher et al. 2015). This pathway is activated in densely confluent epithelial cell cultures and inactivated when cell density is sparse, allowing cells to spread across the substrate. In these situations, the transcriptional activator YAP is mainly located in the cytoplasm or nucleus, respectively (Aragona et al. 2013; Zhao et al. 2007). Knockdown of αII-spectrin and βII-spectrin prevents retention of YAP in the cytoplasm in high density cultures (Fletcher et al. 2015). Since knockdown of αII- and βII-spectrin did not have an effect on the localization of YAP under our conditions (sparse cell density on a soft or stiff substrate), we postulate that under these conditions, the localization of YAP is mainly regulated by Hippo-independent mechanisms, including actin polymerization and Smad shuttling (Dupont et al. 2011; Zhao et al. 2007). Our data suggest that spectrins are, in contrast to their crucial role in the Hippo pathway to regulate YAP, not required to regulate fibroblast YAP in the mechanotransduction pathway that acts parallel to the Hippo pathway.

Fibroblasts and more specifically myofibroblasts, are at the heart of fibrosis (Hinz 2010). Fibroblasts undergo major morphological changes when are they activated into myofibroblasts by, e.g., TGFβ1 and changes occur in tissue stiffening during the fibrotic process (Chia et al. 2012; Hinz 2009; Huang et al. 2012). We therefore questioned whether spectrins play a role in the myofibroblast phenotypical switch. We found that knockdown of spectrins did not affect myofibroblast formation, nor did we observe changes in the organization of αSMA stress fibers. Additionally, we found that focal adhesion assembly was unaffected by spectrin deficiency. The finding that the function of myofibroblasts without αII- and βII-spectrin seems unchanged is illustrated by the observation that collagen type I mRNA expression and protein deposition are unaffected, together with unaffected wound closure. These results were unexpected, because it has been shown that knockdown of βII-spectrin leads to the disruption of TGFβ1 signaling as mediated by SMAD proteins (Kitisin et al. 2007; Lim et al. 2014; Munoz et al. 2014; Thenappan et al. 2011). TGFβ1 signaling via SMAD proteins is key for the induction of the myofibroblast phenotypical shift and collagen production (Piersma et al. 2015a). However, these studies primarily focused on the epithelial lineage and mainly in the context of embryonic mouse development (Kitisin et al. 2007; Lim et al. 2014; Munoz et al. 2014; Thenappan et al. 2011). Few studies have focused on the role of spectrins in fibrosis (Mishra et al. 2004; Wang et al. 2012); however, in a model of CCl_4_-induced hepatic fibrosis, βII-spectrin was found to be upregulated (Wang et al. 2012) in contrast to our findings. Fibroblasts derived from different anatomical sites arise from distinct developmental origins (Lynch et al. 2018), and even within an organ, multiple sub lineages of fibroblasts exist and may explain the differences in spectrin function. Along these lines, it is unknown whether hepatic stellate cells are derived from the embryonic endoderm or mesoderm lineage (Yin et al. 2013).

Since we were intrigued by our observations, we also investigated endogenous gene expression of *SPTBN1* when fibroblasts were stimulated with TGFβ1 and noted a fourfold reduction in *SPBTN1* mRNA levels. This suggests that in adult human dermal myofibroblasts, βII-spectrin does not interfere with SMAD-mediated gene expression, which is confirmed by our siRNA data, where *SPTBN1* levels are reduced more than 20-fold in combination with TGFβ1 without seeing an effect on collagen production or αSMA formation. The latter suggests that in fibroblasts, downregulation of αII- and βII-spectrin is a pre-requisite for the myofibroblast phenotype switch. Additionally, we studied possible compensatory mechanisms after spectrin knock down but found that loss of αII- and βII-spectrin decreased expression of alternative spectrins. These data suggest that either the expression of various spectrins is tightly linked, or that the siRNA used in this study may have off-target effects. In either case, we demonstrated that loss of spectrins is not required for the acquisition of a dermal myofibroblast phenotype.

In conclusion, αII- and βII-spectrin do not regulate cell adhesion, cell size and YAP localization in human dermal fibroblasts and are not required for the dermal myofibroblast phenotypical switch.

## Electronic supplementary material

Supplemental Fig 1 Knock down of αII- and βII-spectrin affects expression of *SPTBN2* and *SPTBN4.* Relative mRNA expression of *SPTBN2*, *SPTBN4* and *SPTBN5* in response to siRNA-mediated knockdown of *SPTAN2* (αII-spectrin) and *SPTBN2* (βII-spectrin). Two-way ANOVA; * *p* < 0.05, ** *p* < 0.01, *** *p* < 0.001. ANOVA, analysis of variance; TGF, transforming growth factor. (GIF 56 kb)

High resolution image (TIF 2947 kb)
