# Supplementary figures and images for: αII-spectrin and βII-spectrin do not affect TGFβ1-induced myofibroblast differentiation
Source: Cell Tissue Res. 2018 May 3;374(1):165–75. doi: 10.1007/s00441-018-2842-x (PMC6132645; doi:10.1007/s00441-018-2842-x)

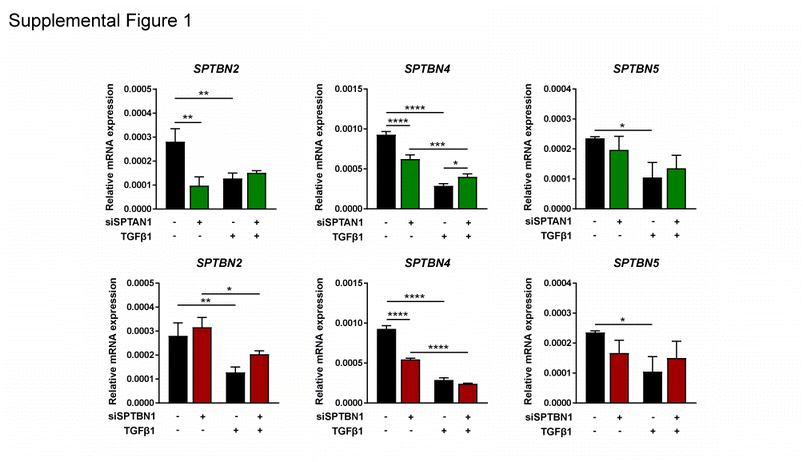

Supplement: Supplementary file 1 — Knock down of αII- and βII-spectrin affects expression of SPTBN2 and SPTBN4. Relative mRNA expression of SPTBN2, SPTBN4 and SPTBN5 in response to siRNA-mediated knockdown of SPTAN2 (αII-spectrin) and SPTBN2 (βII-spectrin). Two-way ANOVA; * p < 0.05, ** p < 0.01, *** p < 0.001. ANOVA, analysis of variance; TGF, transforming growth factor. (GIF 56 kb) [file 441_2018_2842_Fig9_ESM.gif]

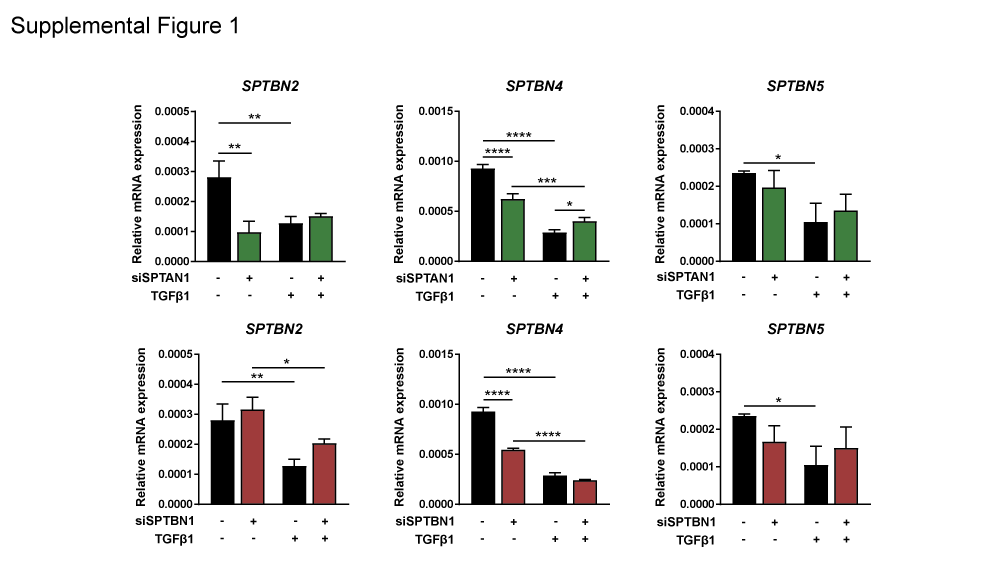

Supplement: Supplementary file 2 — High resolution image (TIF 2947 kb) [file 441_2018_2842_MOESM1_ESM.tif]
